# Auto-enrolment of free school meals: a ‘No Brainer’?

**DOI:** 10.1017/S1368980025000382

**Published:** 2025-03-26

**Authors:** Maria Bryant, Rob Oxley, Myles Bremner, Cressida Pidgeon, Sundus Mahdi, Shona Goudie, Bob Doherty

**Affiliations:** 1 Hull York Medical School, University of York, York YO10 5DD, UK; 2 Department of Health Sciences, University of York, York YO105DD, UK; 3 Bremner & Co, Kineton CV35 0LS, UK; 4 The Food Foundation, London SW9 7QD, UK; 5 School for Business and Society, University of York, York YO10 5DD, UK

**Keywords:** School food, Free school meals, Implementation, Policy, Deprivation

It has been estimated that at least 250 000 children who are entitled to receive a free school meal (FSM) in England are not registered to do so^(1)^. There are many complex reasons why this might be the case, but research indicates issues related to shame, perceived stigma and substantial administrative burden with the FSM application process (influenced by language, literacy and access barriers)^(2,3)^. To qualify for FSM in England, children must live in households where income is below £7400 before tax and benefit support. Thus, children who do not receive their FSM entitlement are living in very high levels of deprivation and missing out on a daily hot meal at school. Importantly, FSM eligibility is also used as a marker of ‘need’ and is linked to ‘Pupil Premium’ funding provided to schools. For every FSM registered child in English primary schools, including special schools, the school receives £1480 (€1754, $1915) per year. For secondary schools, including special schools, this funding equates to £1050 (€1244, $1358)^(4)^. Our ongoing research exploring the benefits and challenges of auto-enrolment processes for FSM (whereby parents are not responsible for applying) is gaining pace. We feel compelled to report on what we are learning along the way; sharing the stories from local governments who are showing us why national auto-enrolment is so critical.

We first learnt of local-level approaches to introducing opt/out, auto-enrolment FSM registration processes from an English local government, ‘Sheffield City council’ (a large city in Northern England) in 2022, where they have been identifying children who are potentially eligible for welfare datasets and applying on their behalf since 2016. In Sheffield, all families whose children are identified as being potentially entitled to FSM from welfare data are contacted to let them know that the council will apply on their behalf unless the family indicates otherwise. In the first year of implementation, Sheffield estimated that they were able to register an additional 1189 children for FSM, which also meant that schools received an additional £1 392 600 (€1 650 168, $1 801 926) in Pupil Premium funding. When we heard this, it felt like a bit of a wake-up call; something that should be done in all areas *and* that could be implemented by the central government. However, we knew we needed solid evidence to back up this policy ask, so we set out to design an evaluation study that could drive informed, evidence-based decisions.

We have been conducting ‘action-oriented’ research since 2023^(5)^; working with local governments in England to develop resources to enable other areas to implement auto-enrolment approaches (which we call ‘the Sheffield approach’). Our research aimed to evaluate both the implementation processes and the impact of auto-enrolment with fifteen local governments. However, we were very quickly inundated with requests to join us and, by the end of 2024, had provided some level of support to seventy-four local governments across England. While all areas are primarily focused on doing the best they can to support families, many (especially those already providing universal FSMs) were also motivated to increase FSM registrations to ensure their schools receive the linked Pupil Premium funding.

Work to secure data on the number of additional pupil registrations and corresponding funding from participating local governments is underway. However, unofficial figures shared via their communication teams are already highlighting the significant impact of auto-enrolment. By October 2024, approximately one-third of areas that we were supporting (*n*-18) had set up auto-enrolment (one as a pilot in just two schools). In seventeen of these areas that have fully implemented the auto-enrolment process, an average of 877 additional children have been registered for FSM per area in the first year of implementation. If we extrapolate these under an assumption that half are primary (and thus providing £648 980 for schools) and half are secondary school children (providing £460 425), this translates into an average of an additional £1 109 405 Pupil Premium funding per area. This estimation is likely to be conservative given that we anticipate that most non-registered children will be in primary schools. If we assume that all seventy-four areas we are currently working with will eventually launch auto-enrolment, extrapolation of early data suggests that approximately 65 000 additional children would be registered from FSM in the first year of implementation, (bringing in approximately £82M in Pupil Premium funding). Of course, we will understand how accurate these figures are when we receive the formal data.

It is important to note that these figures are based on first-year implementation data and may decrease following initial implementation of auto-enrolment. However, while we expected the number of identified pupils to decrease following initial implementation, data suggest that this reduction may be smaller than we originally anticipated. For example, based on data from two areas, on average 1191 pupils were identified during the first year, followed by 837 in the second, highlighting the continued need to implement processes each year. Moreover, one area has observed a year-on-year increase in the identification of children since implementing auto-enrolment, further emphasising the need for ongoing processes.

Perhaps just as compelling are the qualitative data that we have gathered from speaking to families, schools, local governments and national experts, which are further supported by our ongoing analysis of 144 auto-enrolment-related documents. The story is already clear – there is a lot of willingness to undertake FSM auto-enrolment processes; however, it is actually far from ‘automated’. Local governments have put in a considerable amount of investment; not just to set up the processes, but to battle through the bureaucracy and legal challenges that they face… ‘“*It feels very much like we are trying to do something underhand when all we are trying to do is support children [to] access a benefit to which they are legitimately entitled*’ [Quote from one local authority representative]. Given this level of local investment, we support advocacy in this area that is pushing for centralised approaches, where national data and processes are able to circumnavigate the need for local governments to battle against bureaucracy^(6–10)^. In an age of ‘efficiency’, surely a centralised approach is a ‘no brainer’?

The need for FSM auto-enrolment would not be negated by the expansion of universal FSM to all children, as many are calling for^(11,12)^. In fact, we would argue that auto-enrolment becomes even more important when all children are provided with FSM, given that the amount of Pupil Premium funding that schools in England receive is based on the number of children they have who are registered to receive means-tested FSM. Universal provision will likely mean that registration for FSM becomes less of a priority for parents/carers when they are getting a FSM anyway.

Through our work in this area, we know that there are many people working hard to do the best they can for children. However, there are multiple, systemic, barriers that still need to be overcome to ensure that school meals are able to fully support the growth and development of children. School food funding is not in keeping with the real price of a meal^(13)^; food quality varies considerably and is not monitored^(14)^ and priorities differ between governors, leaders, parents and children^(15)^. We know that there is still plenty to do to ensure meals are of a high nutritional quality, whilst being feasible to procure, simple to cook and desirable to children (whilst also limiting environmental harm). However, there is also compelling evidence that having a school meal provides better nutrition than packed lunches^(16)^. This is especially important for children living in areas of high deprivation, who are at greater risk of food insecurity and whose diets are of poorer nutritional quality than those in more fortunate circumstances^(17)^. National data indicates that children on FSM get most of their recommended intakes of nutrients at school^(18)^. There is also growing evidence of the economic benefits of school meals^(12)^, both to the families and the wider economy. FSM improve educational outcomes, leading to better productivity, higher lifetime earnings and greater contributions to the economy. They help to reduce healthcare costs linked to diet-related illnesses. The initiative also alleviates financial pressure on families and stimulates local economies by increasing demand in the food industry^(12,19)^.

Since we started working in this area, the conversation has gathered pace in the UK, with many members of parliament (MP) and other decision-makers advocating for a centralised process to automatically register entitled children for FSM rather than putting the burden on local governments^(7–10,20)^. The subject has been debated in the House of Commons (9th December 2024, 16th October 2024) and has been proposed within multiple policy recommendations, including the 2024 House of Lords Evidence Select report on Food, Diet and Obesity^(9)^. Yet, despite this clear need to consider ways to enhance the system to ensure those who are entitled receive benefits, alongside growing evidence (including our FSM evidence), there remains a reluctance to push this forward by central and senior decision makers. Key to this is the notion of devolution, where the central government has transferred the power and funding from national to local governments in England. We agree this approach ensures that policies can be more relevant to local communities. However, it should not be used as a way to devolve responsibility when the evidence points towards the need for central control. In the case of FSM, the data that we are gathering consistently tells us that local governments are leading auto-enrolment processes to support their children, families and schools, despite the challenges and barriers that they face. National implementation of the approach aligns with the UK Government mission of breaking down barriers to opportunities. Changing to a centralised system will require careful consideration of data-sharing processes; however, it does not equate to a new welfare policy requesting more funding from the Treasury. Provided budgets are estimated appropriately based on entitlement. It feels like it should be a relatively easy win that makes a difference to families and children in greatest need.
